# The Bacterial Diseases of Respiration, and Vaccines in Their Treatment

**Published:** 1913-09

**Authors:** 


**The Bacterial Diseases of Respiration, and Vaccines in their Treatment.** By R. W. Allen, M.D., B.S. Pp. x, 236. London: H. K. Lewis. 1913. 6s. net.

This book is largely a re-presentment of articles by the author which have appeared in the *Journal of Vaccine Therapy* during the last twelve months. It is therefore an up-to-date exposition of the author's views and practice. It contains a detailed description of the various organisms found in the respiratory tract, both pathogenic and non-pathogenic, with directions for obtaining and examining pathological products from the bacteriological point of view. The vaccine treatment of the various diseases of the respiratory organs is very fully dealt with, and the last two chapters are devoted to the diagnosis and specific treatment of pulmonary tuberculosis.

The book deals with subjects which are to a considerable extent controversial, at any rate as to detail, and which require for their appreciation a large experience and acquaintance with authoritative opinion. It will be found of interest to those practitioners who have specially studied the subjects of which it treats, although they may not always be able to accept the author's views in their entirety.

There are a number of photographs and micro-photographs illustrating bacterial growths, but we doubt whether the latter will prove of much assistance to those previously unacquainted with the morphology of the organisms depicted.

